# Changes in brain perfusion with training-related visuomotor improvement in MS

**DOI:** 10.3389/fnmol.2023.1270393

**Published:** 2023-11-09

**Authors:** Eleonora Patitucci, Ilona Lipp, Rachael Cecilia Stickland, Richard G. Wise, Valentina Tomassini

**Affiliations:** ^1^Cardiff University Brain Research Imaging Centre (CUBRIC), School of Psychology, Cardiff, United Kingdom; ^2^Max Planck Institute for Human Cognitive and Brain Sciences, Leipzig, Germany; ^3^Institute for Advanced Biomedical Technologies, University of Chieti-Pescara “G. d’Annunzio,” Chieti, Italy; ^4^Department of Neurosciences, Imaging and Clinical Sciences, University of Chieti-Pescara “G. d’Annunzio,” Chieti, Italy; ^5^Division of Psychological Medicine and Clinical Neurosciences, Cardiff University School of Medicine, Cardiff, United Kingdom; ^6^Helen Durham Centre for Neuroinflammation, University Hospital of Wales, Cardiff, United Kingdom

**Keywords:** multiple sclerosis, MRI, perfusion, training, plasticity, recovery

## Abstract

Multiple sclerosis (MS) is a chronic inflammatory disease of the central nervous system. A better understanding of the mechanisms supporting brain plasticity in MS would help to develop targeted interventions to promote recovery. A total of 29 MS patients and 19 healthy volunteers underwent clinical assessment and multi-modal MRI acquisition [fMRI during serial reaction time task (SRT), DWI, T1w structural scans and ASL of resting perfusion] at baseline and after 4-weeks of SRT training. Reduction of functional hyperactivation was observed in MS patients following the training, shown by the stronger reduction of the BOLD response during task execution compared to healthy volunteers. The functional reorganization was accompanied by a positive correlation between improvements in task accuracy and the change in resting perfusion after 4 weeks’ training in right angular and supramarginal gyri in MS patients. No longitudinal changes in WM and GM measures and no correlation between task performance improvements and brain structure were observed in MS patients. Our results highlight a potential role for CBF as an early marker of plasticity, in terms of functional (cortical reorganization) and behavioral (performance improvement) changes in MS patients that may help to guide future interventions that exploit preserved plasticity mechanisms.

## Introduction

In multiple sclerosis (MS), the modulation exerted by pro-inflammatory mediators on synaptic plasticity might negatively affect the plastic potential of the brain (Di Filippo et al., 2008) and thus its recovery from damage. Nevertheless, Reddy et al. (2000) demonstrated that the ability for systems-level functional reorganization of the motor circuits is preserved in MS brains despite the presence of severe inflammation. Moreover, cortical reorganization has been associated with improvements in motor function (Tomassini et al., 2012b). It is desirable that neuroplasticity be understood and exploited to promote functional and structural reorganization through targeted training interventions.

MRI techniques have been employed to investigate neuroplasticity in MS. Structural MRI studies have demonstrated the effect of 8–12 weeks of motor training, including motor rehabilitation (Bonzano et al., 2014), balance training (Prosperini et al., 2014) and physiotherapy (Ibrahim et al., 2011), on white matter microstructure. FMRI has been able to detect brain functional reorganization in MS in the resting state (Roosendaal et al., 2009); furthermore, shifts in inter-hemispheric lateralization and the recruitment of additional brain areas were reported with the use of motor tasks (Pantano et al., 2002; Rocca et al., 2005; Wegner et al., 2008; Petsas et al., 2013). Changes in patterns of brain activity following visuomotor training (Tomassini et al., 2012b,^2016^) and cognitive rehabilitation (Filippi et al., 2012) have also been reported in MS patients. Taken together, these studies show that neuroinflammation does not completely hinder neuroplasticity. However, it may favor altered mechanisms of neuroplasticity compared to healthy brain (Morgen et al., 2004).

Blood oxygenation level dependent (BOLD) fMRI is the most applied neuroimaging technique to evaluate neuroplasticity related changes in brain activity during the performance of a task. It provides good sensitivity to the hemodynamic changes arising from varying brain activity over a few tens of seconds. However, BOLD fMRI is not suited to measuring longer-term changes in the activity state of brain tissue, for example, over days or weeks. Cerebral Blood Flow (CBF, ml/100 g of tissue/minute), measured non-invasively using the technique of arterial spin labeling (ASL) (Buxton et al., 1998; Liu and Brown, 2007) offers a more stable measure of the basal activity state of brain tissue over long timescales, under the assumption of preserved coupling between neural activity and cerebral blood flow (CBF). It is, therefore, adapted to studying the hemodynamic effects of training interventions aimed at inducing plasticity.

While training-related CBF changes have been studied less than structural changes in the brain, examples of training-related alterations in CBF are suggestive of a facilitatory role of increasing CBF (Smith et al., 2010; Chapman et al., 2013; Maass et al., 2015; Alfini et al., 2019). It is plausible that plasticity of blood supply, or the increase in CBF associated with a chronic increase in neuronal activity, may be an indicator of plasticity and a precursor to longer term structural changes in brain tissue (Mozolic et al., 2010).

Moreover, changes in cerebrovascular function, including a widespread reduction in CBF, may contribute to the pathophysiology of MS (D’haeseleer et al., 2011; Zlokovic, 2011). Metabolic impairment may contribute to hypoxia, demyelination, neuronal loss, brain atrophy and disability progression (Lucchinetti et al., 2000; Lassmann, 2003; Wuerfel et al., 2004; Ge et al., 2005; Trapp and Stys, 2009; Marrie et al., 2010; Holland et al., 2012; Juurlink, 2013; Debernard et al., 2014; Kappus et al., 2016). Previous studies have reported that when the structural brain damage becomes detectable (in terms of GM atrophy and network disruptions), the cognitive impairment in MS is also present (Fleischer et al., 2019; Nauta et al., 2021), suggesting that structural changes may be a subsequent outcome of the cerebrovascular impairment and CBF may thus offer an earlier marker of neuroplasticity. Therefore, inducing an increase in CBF through sustained training on a task may combat some of these potential mechanisms of damage and provide a target for improving tissue physiological status.

The present study employs a multi-modal structural and functional MRI approach, including CBF measurement as a marker of tissue energy supply, to explore longitudinal brain changes that could underlie and support brain reorganization in MS and are associated with a visuomotor sequence learning task. Such tasks have been extensively used in MS as standardized, experimental probes of functional recovery that exploit mechanisms of neuroplasticity (Ghilardi et al., 2009; Zeller and Classen, 2014; Zahiri et al., 2017), given their capacity to induce changes of functional activity in brain areas engaged in the task, measurable through fMRI (Mancini et al., 2009).

At baseline, MS patients and controls were studied during the execution of a sequence learning task and at rest during structural and microstructural (diffusion weighted) MRI, and measurement of CBF. Participants were then trained on a visuomotor task for 4 weeks, in order to investigate mechanisms supporting functional reorganization; longitudinal functional changes were then associated with performance improvements, changes in the vascular state of the brain (CBF), and structural and microstructural characteristics of brain tissue.

## Materials and methods

### Participants and study design

We recruited patients with a diagnosis of Relapsing-Remitting MS (Polman et al., 2011), who fulfilled the following eligibility criteria: age between 18 and 60 years, right-handed, retained use of their right upper limb, no relapse or change in treatment for at least 3 months before study entry, no other neurological or psychiatric conditions. Nineteen age- and sex-matched controls were also recruited. All the participants are a subset of the cohort from the already published study by Lipp et al. (2020a) who consented to a follow-up assessment.

Participants underwent a baseline assessment of demographic, clinical and behavioral measures, as well as multi-modal MRI data. Then, they were asked to practise a serial reaction time (SRT) task at home for 4 weeks and to subsequently return for the second behavioral and MRI assessment.

The study was approved by the UK NHS South-West Ethics Committee (reference: 15/SW/0105) and the Cardiff and Vale University Health Board Research and Development. All participants provided written informed consent.

To investigate between-group differences in age and behavioral measures, we used a two-tailed unpaired *t*-test. A chi-square test was used to assess sex differences between groups. For all the statistical tests, differences were considered significant at *p* ≤ 0.05. Values are reported as mean ± standard deviation (SD), unless stated otherwise.

## Visuomotor training

### Serial reaction time (SRT) task

We used a SRT task to probe recovery experimentally (Tomassini et al., 2011, 2012b; Tacchino et al., 2014; Lipp et al., 2020a). Participants were asked to respond to the location of visual stimuli presented on a computer screen as quickly as possible by pressing the corresponding key on a keypad with one of four fingers (index to little finger) of their right hand.

For the home training, stimuli were presented as a repetitive sequence (Sequence condition) or in a pseudorandom order (Random condition). The Sequence condition included 14 blocks, each consisting of three repeats of 16 stimuli. The sequence was matched in difficulty to the pseudorandom stimuli. After every two Sequence blocks, a Random block was presented. There was a 10 s rest period between blocks. Stimulus duration was 325 ms and the inter-stimulus interval was on average 175 ms (between 150 and 200 ms for each trial). Participants were asked to practice the SRT task on a laptop for 15 min daily, for 5 days per week, for a total of 4 weeks. As part of their home practice, participants were given guidance to complete a paper-based practice log sheet, and the study team conducted weekly phone calls to monitor compliance. The participant’ responses to the tasks were automatically recorded on the laptop as they performed the task.

**FIGURE 1:**
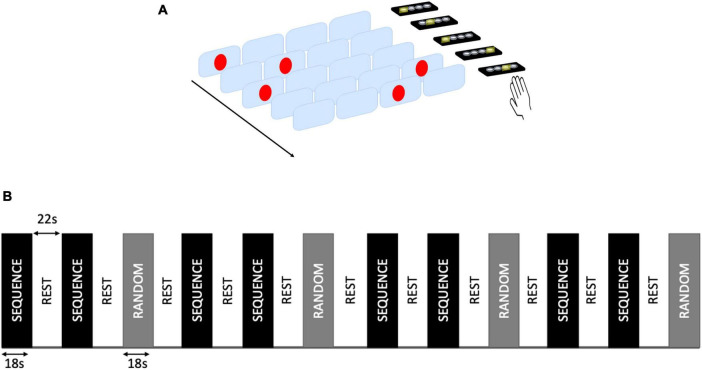
Serial reaction time (SRT) task. **(A)** Example of SRT task presented during the scan. Participants were asked to respond as quickly as possible to the location of visual stimuli presented on a computer screen by pressing the corresponding key on a keypad with one of four fingers (index to little finger) of their right hand. **(B)** Block design of the task. A total of 12 task blocks (8 Sequence and 4 Random blocks) were interleaved with rest blocks.

A version of SRT task was also presented in the scanner, both at baseline and at week 4. The task included a total of 12 blocks (8 of Sequence condition and 4 of Random condition, interleaved with Rest blocks). Stimulus duration was 325 ms and inter-stimulus interval was 175 ms. Responses were given with the right hand on a keypad (Figure 1). All the subjects completed the home training and the task presented in the scanner.

### Training measures

In order to establish whether participants’ performance had changed with the training, for each of the two scan sessions, average accuracy (number of correct responses) and RT across all responses within sequences blocks were calculated for each participant. A two-way ANOVA was performed to investigate changes in accuracy and RT after the training and between-group differences.

To quantify the changes in performance over days of practice, the slope of improvement over time was calculated from the mean of correct responses (accuracy) and median latency (RT) for each subject and for each home training session of the SRT task on sequence blocks only.

## Brain MRI

### Structural characteristics

*In vivo* MRI data were acquired on a 3T General Electric HDx MRI system with an eight channel receive-only head RF coil (GE Medical Systems, Milwaukee, WI). We acquired high-resolution 3D Fast Spoiled Gradient-Recalled-Echo T1-weighted images (resolution = 1 mm × 1 mm × 1 mm, matrix size = 256 × 256 × 172, TE = 3 ms, TR = 7.5 ms, flip angle = 20°, 7.5 min) for gray matter (GM) volumetric analysis and for co-registration of all MRI modalities. We also acquired T2-weighted images [proton-density/T2-weighted dual echo sequence, resolution = 0.94 mm × 0.94 mm × 4.5 mm, 36 slices (3 + 1.5 mm gap), TEs = 9.0/80.6 ms, TR = 3000 ms, flip angle = 90°] and T2-weighted fluid-attenuated inversion recovery sequence (resolution = 0.86 mm × 0.86 mm × 2.4 mm, matrix size = 256 × 256, 36 slices, 3 mm + 1.5 mm gap, TEs = 122.3 ms, TR = 9502 ms, TI = 2,250, flip angle = 90°) for identification of white matter lesions.

### Functional MRI

The short version of the SRT task was presented in the scanner while BOLD-weighted functional MRI (fMRI) images were acquired (resolution = 3.4 mm × 3.4 mm × 3 mm, TR = 3000 ms, TE = 35 ms, FOV/slice = 220 mm, flip angle = 90°, 46 slices of 3 mm with a 1 mm slice gap acquired in AC-PC orientation and an interleaved order, 142 volumes, duration 7 min).

### Cerebral blood flow

To quantify resting cerebral blood flow (CBF) we used multi-inversion time pulsed arterial spin labeling (ASL). We employed a multi-inversion time PICORE QUIPSS II sequence with a dual-echo gradient-echo readout and spiral k-space acquisition (Warnert et al., 2015) (resolution 3 mm × 3 mm in plane, 22 slices, 7-mm thickness with 1 mm gap, TE_1_ = 3 ms; TE_2_ = 29 ms; TR = 4 s), and with the saturation pulse for time-defined bolus set at 700 ms. Four inversion times were acquired (TI = 1100, 1400, 1700, and 2000 ms), each collecting eight signal averages to increase the signal to noise ratio. A M_0_ image with the same resolution as the ASL data was acquired for calibration purposes. A minimal contrast image was acquired with TE = 11 ms, TR = 2000 ms to correct for the coil sensitivity profile.

### Diffusion weighted imaging

Whole brain diffusion weighted images were also acquired with a twice-refocussed diffusion-weighted sequence (40 uniformly distributed directions, *b* = 1200 s/mm^2^) and six non-diffusion weighted images at the beginning (resolution: 1.8 mm × 1.8 mm × 2.4 mm, 57 slices, TE = 94.5 ms, TR = 16000 ms, flip angle = 90°). Fifty-seven contiguous axial slices were acquired with a field-of-view of 230 mm × 230 mm, acquisition matrix of 96 × 96, giving an isotropic acquisition voxel dimension of 1.8 mm.

## Data analysis

### Lesion identification

White matter lesions were identified by an experienced reader using the software package JIM (v.6, Xinapse System, Leicester, England) on T2-weighted images, consulting the PD-weighted images and the T2-weighted fluid-attenuated inversion recovery (FLAIR) sequence.

For lesion filling of the T1-weighted image, the T2-weighted image was registered to the high- resolution T1-weighted image with an affine registration using FLIRT (Jenkinson et al., 2002). The resulting registration matrix was applied to the T2-derived lesion maps. The resulting interpolated lesion map was thresholded at 0.5 to approximately preserve the size of the original lesion map, but also to allow a small amount of inflation in order for the lesion map to better overlap with the lesions on the T1-image, in case of registration errors, and it was then binarized. Lesion volume was calculated for each patient (Battaglini et al., 2012; Gelineau-Morel et al., 2012). FSL FAST (Zhang et al., 2001) was used to create a white matter probability map for the T1-weighted image. Using FSL’s lesion filling tool [“lesion_filling” (Battaglini et al., 2012)], we filled the lesion areas with intensities similar to those in the non-lesion neighborhood. FSL-FAST was then run again on the lesion-filled T1-image to produce a robust PVE of GM.

### Structural image analysis

To test for localized differences between groups in gray matter volume at both time points and longitudinal differences within the group, T1 weighted images were analyzed with FSL-VBM (Good et al., 2001).^1^ A voxel-wise GLM (General Linear Model) was applied using permutation-based non-parametric testing, correcting for multiple comparisons across space using threshold-free cluster enhancement (TFCE) (Smith and Nichols, 2009). Voxel-wise GLM was also applied using permutation-based non-parametric testing to investigate the correlation between changes in GM and the slope of improving of behavioral performance. Differences were considered significant if *p* < 0.05. Brain tissue volume, normalized for subject head size, was estimated with SIENAX (Smith et al., 2002), part of FSL.

### Microstructural analysis

DWI data were pre-processed by using the FMRIB’s Diffusion Toolbox (FDT) (Smith et al., 2004). After correction for head motion and image distortion due to eddy currents, a diffusion tensor model was fitted at each voxel and fractional anisotropy (FA) maps were obtained (Lipp et al., 2020b). A voxel-wise statistical analysis of the FA data was carried out using tract-based spatial statistic (TBSS) (Smith et al., 2006) to investigate longitudinal changes in brain microstructure with training. For each participant we linearly registered the FA maps from both scanning sessions to a space mid-way, using the registration matrix from the respective T1-weighted image to the mid-space created by SIENA, to avoid bias of the skeleton toward one of the two sessions. We then averaged the two co-registered maps for each participant to create a subject specific mid-space template. All the subject-specific templates were then aligned to the FSL standard template and averaged to create a study specific mid-space mean FA map. For each subject, we projected local tract centers onto the skeleton of the mid-space mean FA map and used these images in the statistical comparisons. Statistical results were considered significant for *p* < 0.05.

### Functional MRI

Analyses were carried out using FEAT (FMRIB Expert Analysis Tool, v6, Oxford University, UK). Pre-processing steps included: skull stripping using BET (Smith, 2002), MCFLIRT motion correction (Jenkinson et al., 2002), high pass filtering (100 s temporal cut off), spatial smoothing with a Gaussian kernel of full-width-half-maximum 5 mm. Functional images were registered using boundary-based registration with simultaneous field map correction (Greve and Fischl, 2010).

To model the task, two conditions (Sequence and Random) were defined, contrasting each condition to Rest. The model was convolved with the hemodynamic response function (a gamma function) and temporal filtering was applied to the model. Temporal derivatives of the event regressors were included as regressors of no interest. One main contrast of interest was defined (sequence >rest) reflecting hemodynamic activation during task performance.

To investigate between group differences in functional activity, we set up an unpaired *t*-test at session 1 for the contrast of interest (sequence >rest), using FSL FLAME1 with outlier de-weighting, a voxel-threshold of *Z* > 2.3 and a two-sided statistical cluster threshold of *p* < 0.05. Gray matter volume was used as regressor of no interest to account for potential effects of differing brain tissue volume. To investigate within group differences between scan sessions and the group × time interaction, we set up a repeated measures mixed model for the contrast sequence >rest, using FLAME1, with a voxel-threshold of *Z* > 2.3 and a two-sided statistical cluster threshold of *p* < 0.05. To investigate the correlation between changes in functional activity during the task and SRT task-related changes in performance, a voxel-wise GLM was applied using permutation-based non-parametric testing. Differences were considered significant if *p* < 0.05. Testing for functional activation was constrained to gray matter regions.

### Blood flow quantification and changes with performance

The M0 calibration image was used to define the spatial transformation from CBF data space to high resolution T1-space, using FLIRT (Jenkinson et al., 2002) with 6 degrees of freedom. CBF maps were estimated using *oxford_asl* with partial volume correction (Chappell et al., 2011), through the application of FAST-derived estimated GM probabilities (Zhang et al., 2001). CBF maps were transformed to the space of the T1-weighted structural scan using the registration matrix mentioned above. Voxel-wise GLM was applied using permutation-based non-parametric testing, correcting for multiple comparisons across space using threshold-free cluster enhancement (TFCE) (Smith and Nichols, 2009), to investigate baseline differences between groups, longitudinal changes in CBF between sessions and the correlation between changes in CBF maps and the slope of improvements in behavioral performance (both with accuracy and reaction times). Differences were considered significant if *p* < 0.05.

## Results

### Baseline demographic and clinical characteristics

Twenty-nine MS patients and 19 matched healthy volunteers were recruited. Participants’ characteristics are shown in Table 1. There was a significant difference in the level of education between groups [*t*_(44)_ = 2.98; *p* = 0.004]. Patients differed from controls in left hand dexterity [*t*_(46)_ = −2.61; *p* = 0.01] and walking ability [*t*_(46)_ = −2.19; *p* = 0.03] and had lower normalized GM brain tissue volume [*t*_(46)_ = 2.46; *p* = 0.02].

**TABLE 1:** Demographic, clinical and MRI characteristics.

	Patients	Controls	*p*
*N*	29	19	–
Age (years)	39.2 ± 11.3	40.5 ± 11.0	0.69
Sex (F/M)	17/12	12/7	0.75
Education (years)	15.9 ± 3.9	19.7 ± 4.8	<0.01*
Disease duration (years)	7.6 ± 4.3	–	–
DMT* (Yes/No)	13/16	–	–
EDSS (median/iqr)	2.4 ± 1.1	–	–
MSIS-29 scale physical	31.6 ± 12.4	–	–
MSIS-29 scale psychological	16.7 ± 6.5	–	–
Depression (BDI)	8.2 ± 8.6	4.6 ± 5.1	0.11
Fatigue (MFIS)	27.7 ± 19.3	21.1 ± 13.3	0.21
9-HPT (right) in sec (across 2 trials) median/iqr	21.7 ± 7.1	18.2 ± 4.7	0.06
9-HPT (left) in sec (across 2 trials) median/iqr	23.3 ± 4.7	19.6 ± 3.8	<0.01*
T25-FW in sec (across 2 trials) median/iqr	5.0 ± 2	4.3 ± 1	0.03*
No. correct responses PASAT 3 s	44.5 ± 12.2	51.0 ± 6.4	0.14
No. correct responses PASAT 2 s	31.1 ± 8.6	33.9 ± 7.1	0.7
SDMT	57.1 ± 11.1	61.4 ± 8.3	0.77
WLG	24.4 ± 7.3	28.3 ± 7.1	0.69
Memory verbal delayed	8.9 ± 2.5	9.8 ± 2.0	0.19
Memory spatial delayed	7.2 ± 2.3	7.8 ± 2.1	0.35
Normalized GM volume (mm^3^)	613.1 ± 47.9	645.8 ± 39.9	0.02*
NBV (mm^3^)	1196.4 ± 109.5	1230.0 ± 87.0	0.27
T2-hyperintense lesion volume (cm^3^)	2.8 ± 2.4	–	–

Unless indicated otherwise, descriptive statistics are reported as means and standard deviations. For statistical comparisons between groups, chi-square was computed for categorical-variables, Kruskall-Wallis test for skewed variables (9-HPT, T25-FW), and unpaired *t*-test was used for other variables. *p*-values were considered significant if *p* < 0.05. PASAT, SDMT, WLG, and normalized GM volume were adjusted for years of education. Normalized brain and gray matter volume were calculated with SIENAX. EDSS, extended disability status scale; MSIS, multiple sclerosis impact scale; DMT, disease modifying treatment; BDI, beck depression inventory; MFIS, modified fatigue impact scale; 9-HPT, 9-hole peg test; T25-FW, timed 25-foot walk; PASAT, paced auditory serial addition test; SDMT, symbol digit modalities test; WLG, word list generation. *List of specific medications, along with the number of patients on each specific DMT: Avonex = 3; Betaferon = 1; Copaxone = 1; Fingolimod = 2; Rebif = 1; Tecfider = 3; Tysabri = 2. Bold values indicate significant statistical difference between groups.

### Behavioral results—SRT task

**FIGURE 2:**
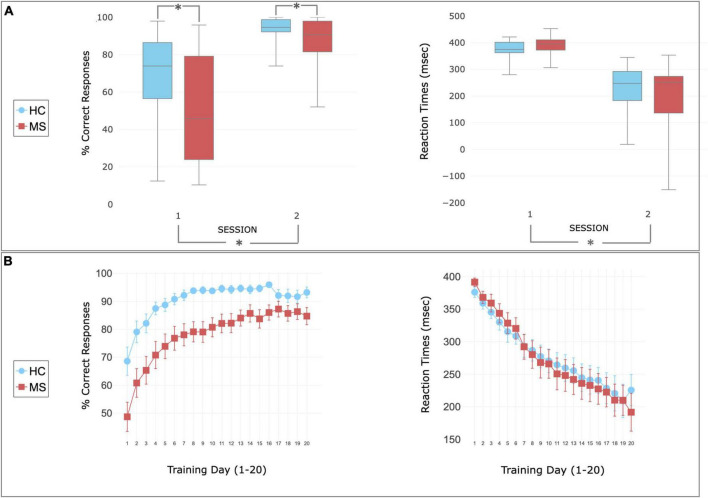
Behavioral performance during SRT task. **(A)** Performance recorded during the scan session 1 (before the home training) and 2 (following the home training). Patients (red) and controls (blue) showed increased accuracy (left) and decreased reaction time (right) after the training. Higher accuracy was observed in HC compared to MS in both sessions. The center of the plot represents median. The bars represent the range from lower and upper quartile. Whiskers indicate variability outside the upper and lower quartiles (maximum and minimum values). Asterisks indicate statistically significant differences between groups (top—MS patients report lower than controls) or sessions (bottom—performance improving after the training). **(B)** Performance recorded during the home training and reported as mean ± sem. Patients (red) and controls (blue) showed increase of accuracy and decrease of reaction times with the home training.

There was higher accuracy (% of correct responses) in controls than in patients in both sessions [*F*_(1,46)_ = 8.3, *p* = 0.006], with no significant group difference in RT [*F*_(1,46)_ = 0.029, *p* = 0.863]. There was an increase in the number of correct responses [*F*_(1,46)_ = 61.81, *p* < 0.01] and a decrease in RT [*F*_(1,46)_ = 118.81, *p* < 0.01] from session 1 to session 2 in both groups, with no group × time interaction for accuracy [*F*_(1,46)_ = 3.22, *p* = 0.08] or for RT [*F*_(1,46)_ = 1.34, *p* = 0.25] (Figure 2).

### Functional activation and changes after training

**FIGURE 3:**
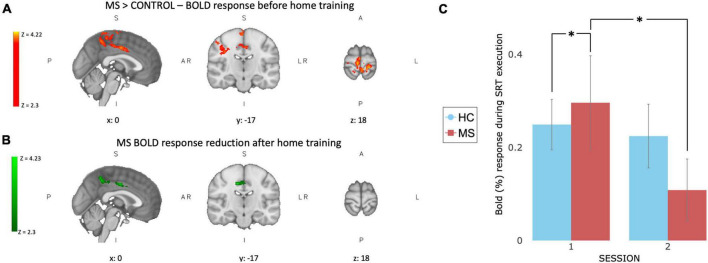
Functional BOLD activation and changes after 4 weeks of training. At baseline **(A)** MS patients show higher task related BOLD activation compared to controls in 4 clusters: right hemisphere, mainly corresponding to the pre-cuneal cortex, left cingulate gyrus and bilaterally in the medial portion of pre and post-central gyri. After 4 weeks of SRT training **(B)** a significant group × time interaction was observed in MS patients in the right pre-central gyrus and the right inferior temporal lobe. **(C)** BOLD response extracted from green ROI, showing a larger reduction in task induced BOLD response after training (session 2) in MS group (red) compared to controls (blue). Values are reported as BOLD% change, error bars represent SEM. *Indicate a significant statistical difference between groups.

At baseline, before home training, patients showed higher task related BOLD signal activation than controls in 4 clusters on the right hemisphere, mainly corresponding to the pre-cuneal cortex, left cingulate gyrus and bilaterally the medial portion of pre and post-central gyri (Figure 3A). After 4 weeks of training a significant group × time interaction was found mainly in the right pre-central gyrus and the right inferior temporal lobe indicating a change in task-induced BOLD signal changes between scan sessions that was different between groups (Figure 3B). This was due to reduced BOLD signal response to the task after the training in MS patients (Figure 3C).

In neither group did longitudinal changes in BOLD responses during task execution correlate with behavioral changes (accuracy or reaction time recorded during the home training).

### Perfusion at baseline and changes with training

**FIGURE 4:**
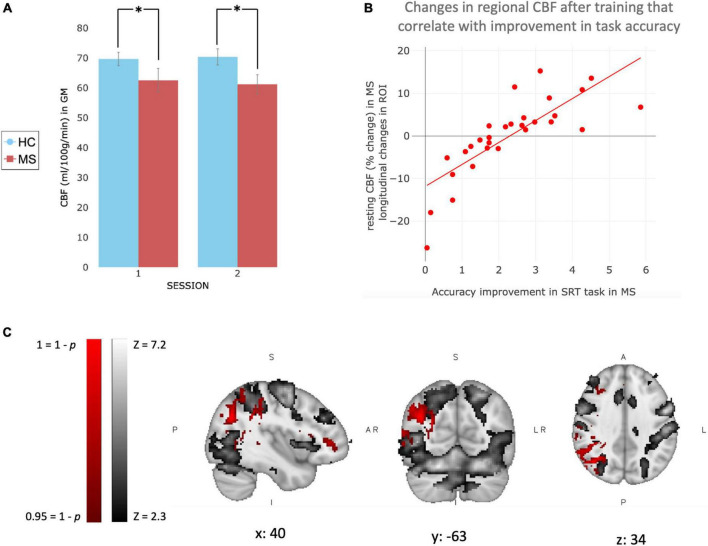
Changes in brain perfusion with training in MS patients. **(A)** Bar graph representing CBF values (ml/100 g/min) extracted in Gray Matter only for MS patients (red) and healthy controls (blue). Values are reported as Mean ± SEM. **(B)** Plot showing the trend of the correlation observed with the voxel-wise analysis. CBF values (percentage change after training compared to before) were extracted only in significant area [red area in **(C)**] and plotted against the increase of performance, calculated by the slope of improvement in accuracy, in MS patients. **(C)** Patients’ mean BOLD response to task at baseline (gray), and voxel-wise correlation between behavioral SRT (accuracy) improvement over home-training and increase in resting perfusion in patients after 4 weeks of training (red). *Indicate a significant statistical difference between groups.

At baseline, voxel-wise analysis reported a significant difference between groups in the resting perfusion of the brain, with healthy volunteers showing higher perfusion in most of the brain. The between-group differences were also present at the second time point (mean ± SEM CBF values extracted in GM: Session 1–HC: 69.64 ± 2.24 ml/100 g/min; MS: 62.49 ± 4.01 ml/100 g/min. Session 2–HC: 70.35 ± 2.69 ml/100 g/min; MS: 61.17 ± 3.19 ml/100 g/min) (Figure 4A).

Voxel-wise analysis was used to investigate longitudinal changes in resting perfusion after 4 weeks of training. We did not observe significant longitudinal changes in the MS or control group. However, voxel-wise analysis showed that after 4 weeks of training, changes in accuracy only (slope of changes) correlated positively with changes in perfusion in the right angular and right supramarginal gyrus in MS only (Figure 4), brain areas that partially overlap with regions activated by the task. The same correlation was not observed for reaction times and it was not observed in healthy controls (neither in reaction times or accuracy).

In order to investigate any potential influence of right upper limb motor disability, we ran a second voxel-wise analysis. This analysis investigated the correlation between changes in accuracy and changes in CBF using the results from 9HPT performed with the right hand, which assess upper limb functionality, as a covariate of no interest.

Voxel-wise analysis with 9HPT as a covariate of no interest, confirmed that in MS after 4 weeks of training, changes in accuracy (slope of changes) were positively correlated with changes in perfusion in the right angular and right supramarginal gyrus. Indicating that the observed changes are not contingent upon the presence of motor disability in the right upper limb.

### Structural MRI

**FIGURE 5:**
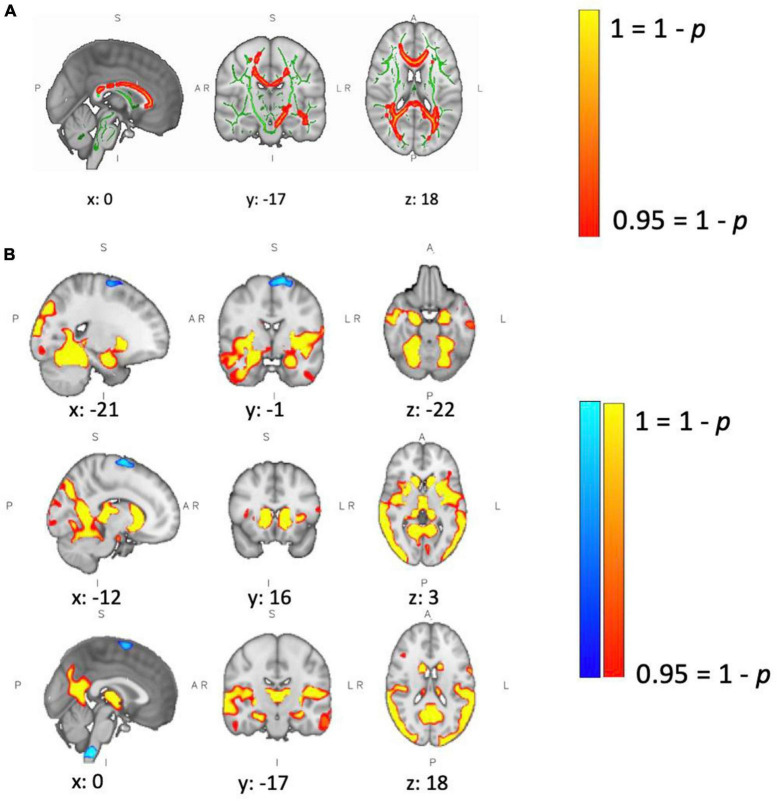
Between-group differences in brain structure. **(A)** Patients showed lower WM fractional anisotropy (FA) at baseline compared to controls. Green defines the WM skeleton, in which the group-based statistical contrast was carried out; yellow-red indicates regions where patients show lower FA than controls (corpus callosum, left and right corticospinal tract, and left and right optic radiation). Differences were considered significant at *p* < 0.05. **(B)** Patients showed lower GM volume at baseline in yellow/red regions, including left/right fusiform gyrus, left/right intra-calcarine gyrus, left/right cingulate gyrus, left/right inferior temporal gyrus, right parahippocampal gyrus, left/right thalamus, left/right caudate, right putamen and cerebellum. Patients showed higher GM volume in brain stem, left frontal gyrus and left motor cortex. Differences were considered significant at *p* < 0.05.

T2-hyperintense lesion volumes are reported in Table 1. At baseline, patients showed lower FA than controls in major white matter tracts, including the corpus callosum, the corticospinal tracts and the optic radiations (Figure 5A). We did not observe a change in white matter microstructure (FA) after 4 weeks of training in MS or HC group. There was a significant difference in GM volume between groups at baseline (see results in Table 1). We observed a widespread lower GM volume in the patients in the fusiform gyri, intra-calcarine gyri, cingulate gyri, inferior temporal gyri, right parahippocampal gyri, thalami, caudate nuclei, right putamen and cerebellum. Patients showed small areas of localized higher GM volume in brainstem, left frontal gyrus and left motor cortex (medial part) (Figure 5B). Differences in GM volume between sessions were found only in controls, who showed a reduction in right amygdala and putamen. In neither group, was there a correlation between performance improvements and microstructural/macrostructural changes with training.

## Discussion

The aim of this study was to explore longitudinal brain and behavioral changes that are associated with 4 weeks of visuomotor training and that could underlie and support functional reorganization in MS patients. Evidence for functional changes after 4 weeks was observed in MS patients in the form of a reduced BOLD response during visuomotor task execution. This change in MS patients was accompanied by a regionally specific correlation between changes in resting baseline CBF and improvements in accuracy of task performance over 4 weeks. Our results highlight the potential importance of resting CBF as a marker for functional (cortical reorganization) and behavioral (performance improvement) changes in MS patients and the potential benefits of interventions that exploit preserved plasticity mechanisms.

### Functional reorganization after training

A reduction of functional activation after 4 weeks of training was observed in the somatosensory network in MS patients, suggesting the occurrence of cortical plasticity as previous studies reported (Tomassini et al., 2012b). The hyperactivation observed at the first time point in patients compared to controls confirms previous findings showing that MS patients differ from healthy volunteers in functional activation (Rocca et al., 2005; Filippi et al., 2012), specifically reporting hyperactivation in ipsi- and contra-lateral brain regions (Tomassini et al., 2016). Here, the hyperactivation was found in the somatosensory network, which is involved in higher-order processes, such as perception, attention, manual dexterity and coordination (Karhu and Tesche, 1999; Disbrow et al., 2000; Hämäläinen et al., 2000). Given the comparable RT between groups at the first time point, the greater involvement of somatosensory network associated with task execution could be interpreted as a compensatory mechanism where higher level of integration is needed to perform the task to a similar level as healthy controls (Beauchamp et al., 2003; Reber, 2013). Further studies are needed to test this hypothesis.

After 4 weeks of training, we observed a reduction of the hyperactivation and an improvement in accuracy and RT. Our results are in line with the well-known hypothesis that decrease in functional activity reflects more efficient information processing (Haier et al., 1992; Pascual et al., 1995; Shadmehr and Holcomb, 1997; Hardwick et al., 2013) and PET data suggest that the reduction is in part due to an increase in baseline blood flow rather than a decrease in actual brain activity (Xiong et al., 2009). Our results are consistent with a prior study showing a reduction in functional activation following a long period of training in MS patients (Morgen et al., 2004), and they accord with the suggestion that structural/microstructural impairments at the level seen in the MS patients do not inhibit the ability to learn motor skills, perhaps because of the usage of compensatory neural resources such as the less damaged cortico-striatal loop during motor learning (Fling et al., 2015).

We suggest that 4 weeks of visuomotor training leads to a normalization of brain activation toward that of controls, which is an expression of preserved brain plasticity in MS patients (Tomassini et al., 2012a), supported by performance improvement.

### Changes in CBF are associated with improvements in performance accuracy

After 4 weeks of visuomotor training we observed a localized correlation between CBF change and performance improvements quantified in terms of response accuracy in MS in areas related to SRT task execution. Specifically, the association was observed in angular gyrus, which is an attentional area involved in processing visual information, and supramarginal gyrus, which is part of the somatosensory cortex and it is involved in perception of space and location of limbs; confirming the well-known role of attentional and control areas during learning (Schiltz et al., 2001; Ungerleider et al., 2002). A correlation was not observed between performance improvements and longitudinal changes in regional task-induced BOLD signal response. We can confidently exclude the possibility that our results have been influenced by the presence of cardiovascular conditions or the use of vasoactive therapies, because individuals with specific medical conditions (including vascular or respiratory conditions, hypotension, hypertension, epilepsy, and psychiatric disorder) were excluded from study participation. Furthermore, it is unlikely that DMTs affect our results, as the DMT taken by patients are not considered vasoactive medications. Furthermore, even if they have any indirect effect on vascular system, it is unlikely that they would significantly influence CBF changes observed over the 4 weeks study. This is because patients had already been taking these medications for at least 3 months at the time of study entry. Our results are consistent with previous findings reporting an increase in resting perfusion and cognitive and/or physical training, although we do not observe a significant average longitudinal increase in CBF. Specifically, CBF measured with PET has been associated with improvements in SRT task performance in motor areas (Grafton et al., 1995; Hazeltine et al., 1997; Honda et al., 1998). It has also been shown that physical exercise has a modulatory effect on CBF (Querido and Sheel, 2007; Barnes, 2015; Smith and Ainslie, 2017) with study reporting increase in CBF and cerebrovascular reactivity following physical activity (Gligoroska and Manchevska, 2012) suggesting the beneficial effect of training on brain metabolism and on the vascular system (Ainslie et al., 2008; Murrell et al., 2013; Steventon et al., 2018). Furthermore, an increase in CBF of 20% has been found following exercise, accompanied by an increase in motor cortex CBF during a finger tapping task (Smith et al., 2010). These results were consistent with Chapman et al. (2013) that reported higher resting CBF in the anterior cingulate region after 12 weeks of exercise training compared to a control group. On the cognitive side, increase in resting regional CBF of the right lateral PFC has been reported after 4 weeks of working memory training (Takeuchi et al., 2012). These results support previous findings that linked training with enhanced vascular health (Desouza et al., 2000; Whelton et al., 2002), accompanied by improved cognitive performance (Colcombe et al., 2004; Erickson et al., 2011).

Although it has been shown that lower resting CBF correlates with lower neuropsychological performances in healthy volunteers (Renke et al., 2022), in HC we did not observe the same significant correlation between changes in task performance and changes in CBF after training. This may be explained in part by a lower baseline variability in the HC task performance thus they had less room to improve on average (Figure 2) and we hypothesize that the improvement was not big enough to be reflected in a brain perfusion change. Patients also showed variable perfusion changes as shown by the correlation plot (Figure 4). MS studies have reported a correlation between the level of perfusion and different cognitive impairment/lesion load (Lapointe et al., 2018); indicating that a perfusion impairment may make it hard to recruit the resources needed to improve task performance.

The observed lower resting perfusion in MS at baseline is in line with previous studies describing metabolic dysfunction in MS patients compared to healthy controls (Ge et al., 2005; Ota et al., 2013; Absinta et al., 2015; Chandler et al., 2023). Lincoln et al. (2022) showed that blood flow can be enhanced in MS lesions, supporting perfusion as a potential therapeutic target for training that aim to recover function in MS. Although the perfusion impairment is well documented in MS population, few studies have investigated its changes with time. Testud et al. (2022) have shown a decrease in perfusion level in people with MS across a 5 years span. Over the much briefer period of observation in the present study we did not observe a longitudinal change in MS in mean resting CBF in the voxel-wise analysis (neither increase or decrease). On the other hand, the absence of decrease in mean resting perfusion together with the persistence of between group differences at the second time point may suggest the hypothesis that the training acted as a protector of the tissue and prevented further metabolic dysfunction. Also our behavioral results show that the patients were able to perform the task in a comparable way to healthy controls at the second time point, following home training, suggesting that our task was relatively easy and a harder task with more room to improve performance may be able to elicit an increase of mean resting CBF.

Hence our results corroborate the hypothesis of CBF as a promising neural marker of brain changes given a specific intervention (Mozolic et al., 2010; Vas et al., 2015), since the training may boost the energy consumption of neural components, like increases in cellular proteins, enzymes and neurotransmitter turnover (Attwell and Laughlin, 2001; Chapman et al., 2015). The lack of a third measurement time point leaves open the question of how long the changed CBF is sustained. Considering that Chapman et al. (2015) demonstrated that CBF increase is maintained higher after training stopped, we hypothesize a preserved CBF response associated with greater performance improvement in the long term, but further studies are needed to test this hypothesis.

### Lack of changes in structural measures and study limitations

Group differences in WM integrity and GM volume were observed at baseline. The reduction of GM volume in MS patients compared to healthy controls indicates the occurrence of a neurodegenerative process. Our result is consistent with previous studies showing that GM atrophy is common in MS and it occurs already in early stages of the disease (Audoin et al., 2010; Popescu et al., 2015). Despite the group differences observed at baseline, after 4 weeks of visuomotor training, we did not observe an increasing in FA or in GM volume in MS patients. Fractional anisotropy (FA) is one of the most common MRI measures employed to assess WM integrity (Basser et al., 1994). Reduced FA is thought to reflect underlying demyelination and axonal loss (Beaulieu et al., 1996; Schmierer et al., 2007) and our results confirm previous findings reporting lower FA value in MS patients (Ciccarelli et al., 2001; Vrenken et al., 2006). Previous longitudinal studies observed structural changes after training, but, unlike our study, the training was longer than 4 weeks (Scholz et al., 2009; Chapman et al., 2015). Particularly, Chapman et al. (2015) investigated structural changes after 6 weeks and 12 weeks of cognitive changes and they reported that the structural changes emerged only at the later assessment.

We observed an unexpected reduction in GM volume in the amygdala and putamen in HC following 4 weeks of training. Although this finding was unexpected, previous research has indicated that structural changes associated with learning are dynamic, with the brain undergoing distinct phase during the learning process. These studies reported an initial increase in GM volume during the early phase of learning, followed by a subsequent decrease in GM volume (Taubert et al., 2010; Sehm et al., 2014).

The phenomenon mirrors well-established functional mechanisms where different neural networks are recruited at different stage of learning (Karni et al., 1995; Floyer-Lea and Matthews, 2005). The authors propose that this decrease in GM volume reflects a physiological adaptive process resulting from changes in network activation needed to accommodate task learning and adaptation (Sehm et al., 2014). Consistent with prior research, the observed decrease in GM volume within the HC group may be attributed to our cohort being in a later stage of learning at the second time point of the study. However, further studies are needed to test this hypothesis.

It is known that brain tissue repair mechanisms and functional reorganization counterbalance the effect of the inflammation and neurodegeneration pathogenic mechanisms responsible of disease progression (Koudriavtseva and Mainero, 2016). Despite the observed longitudinal changes in functional and metabolic parameters, we did not observe structural longitudinal changes in terms of worsening/recovering of white and gray matter damage in MS. Again, we should also consider the hypothesis that the training acted as a protector of the tissue and prevented further damage (Bonzano et al., 2014), although the time-scale of the training may be too short to detect this change.

Overall, it is noteworthy noting that studies that have recruited different MS phenotypes to examine brain physiology in MS, have treated the cohort as a unified sample, rather than investigating physiological differences across phenotypes (Ota et al., 2013). This oversight persists despite the fact that previous studies have reported differences across different MS population as distinct cognitive profiles (Huijbregts et al., 2004), structural MRI measures (Rocca et al., 2012), disability levels (Antel et al., 2012), and histopathology (Antel et al., 2012). Therefore, further studies investigating different metabolic and physiological profiles are essential to be able to generalize our findings to other MS phenotypes.

### Relationship between resting CBF and structural plasticity

Structural plasticity incorporates many different processes, such as changes in synapse numbers, axonal fiber densities, synaptic connectivity patterns and neuronal cell numbers (Butz et al., 2009). Persistent changes in neural activity can lead to adaptations that include a remodeling of the vascular and neuronal architecture (Lacoste et al., 2014), specifically learning tasks lead to an increase of synapses per unit area (Black et al., 1990; Riddle et al., 1993). These changes require additional energy and thus may be expected to demand a greater blood supply. Furthermore, increase in cerebral blood volume, which positively correlates with CBF after training, correlated with neurogenesis and angiogenesis (Pereira et al., 2007). Therefore, CBF changes may be an early consequence the neuroplasticity process.

The biological mechanisms responsible for these processes are thought to be Insulin-Like Growth Factor-1 (IGF-1) and Brain-Derived Neurotrophic Factor (BDNF). IGF-1 is increased by repeated neural activity following a stimulation, such as during training, and its activity leads to increase of BDNF expression (Cavaglia et al., 2001). BDNF is known to enhance neuroplasticity via different pathways such as synaptogenesis, neurogenesis, and long-term potentiation (Cotman et al., 2007; Muller et al., 2020). BDNF secretion is under the control of neuronal activity-dependent mechanisms (Marie et al., 2018). Then endothelial BDNF controls neuroplasticity through astrocyte-dependent mechanisms (Alderson et al., 2000; Colombo and Farina, 2012) and through activation of tRKb receptors (Rose et al., 2003) resulting in sustained Nitric Oxide (NO) production (Meuchel et al., 2011)which would induce long term potentiation (LTP) (Malenka and Bear, 2004; Hopper and Garthwaite, 2006). NO signaling plays a major role in the regulation of CBF (Garry et al., 2015) and NO formed via endothelial cells causes vasodilation and blood flow increase (Gordon et al., 2007).

Taken together with our lack of changes in WM measures, these results support the hypothesis that structural changes take longer than functional and vascular changes to occur (Bruel-Jungerman et al., 2007) and/or to be detected by MRI measurements. The changes in perfusion observed after the training in MS may be reflected in structural changes in a longer term.

## Conclusion

Our results suggest that the functional reorganization observed in MS patients following a behavioral intervention is associated with a correlation between changes in baseline perfusion and performance improvement. Local alterations in CBF may precede structural and microstructural changes (Wuerfel et al., 2004). Imaging CBF has a potential role as a biomarker for training-related changes and may offer a probe to investigate the mechanisms of plasticity in MS that underlie rehabilitative interventions.

## Data availability statement

The raw data supporting the conclusions of this article will be made available by the authors, without undue reservation.

## Ethics statement

The studies involving humans were approved by the UK NHS South-West Ethics Committee (reference: 15/SW/0105) and the Cardiff and Vale University Health Board Research and Development. The studies were conducted in accordance with the local legislation and institutional requirements. The participants provided their written informed consent to participate in this study.

## Author contributions

EP: Conceptualization, Formal analysis, Investigation, Writing – original draft, Writing – review and editing. IL: Conceptualization, Data curation, Methodology, Writing – review and editing. RS: Data curation, Methodology, Writing – review and editing. RW: Funding acquisition, Supervision, Writing – review and editing. VT: Conceptualization, Funding acquisition, Supervision, Writing – review and editing.

## Footnotes

1
http://fsl.fmrib.ox.ac.uk/fsl/fslwiki/FSLVBM
